# Editorial: Axonopathy in Neurodegenerative Disease

**DOI:** 10.3389/fnins.2018.00769

**Published:** 2018-10-23

**Authors:** Robert W. Burgess, Samuel D. Crish

**Affiliations:** ^1^The Jackson Laboratory, Bar Harbor, ME, United States; ^2^Department of Pharmaceutical Sciences, Northeast Ohio Medical University, Rootstown, OH, United States

**Keywords:** axon degeneration, neuropathy, glaucoma, mitochondrial dysfuncion, chemotherapy induced neuropathy

Within the study of neurodegenerative diseases, axonopathy is increasingly recognized as a major contributor to the disease manifestation, and in some cases, the key pathogenic driver. Neurons, with their highly specialized anatomy, must maintain both dendritic arbors and long axon processes. The loss of the axon effectively disconnects the neuron from its postsynaptic partners, eliminating its function in neural circuitry. The loss of the axon may be an early symptom of more extensive degeneration and neuron loss to follow. This has been suggested for diseases such as amyotrophic lateral sclerosis (ALS), where changes in the distal terminal of motor neurons presage their eventual death (Fischer et al., 2004). In other cases, such as inherited peripheral neuropathies, the loss of the axon is the primary feature of the disease, with sensory and motor deficits manifesting in the distal extremities innervated by the longest neuronal projections (Cavanagh, 1964).

Many aspects of cell biology converge in maintaining axons and in their degeneration. Their length requires an elaborate cytoskeleton and transport machinery. Functioning mitochondria are required to meet local energy demands, again necessitating mitochondrial transport, fusion, fission, and possibly local translation of mitochondrial components to keep up with organelle and protein turnover (Misgeld and Schwarz, 2017). Finally, axons are also often intimately associated with myelinating glial cells, creating a mutually dependent functional unit to maintain axonal conduction and integrity. This Research Topic of Frontiers in Neuroscience is devoted to these issues of axon cell biology and the disorders that result from dysfunction in these processes. Many of the papers included in this Research Topic arose from presentations and discussions held at the 6th Molecular Mechanisms of Axon Degeneration conference, hosted at The Jackson Laboratory in Bar Harbor, Maine, in September of 2016. Additional contributions expand the scope of this Research Topic, particularly into areas related to degeneration of the optic nerve, relevant to glaucoma and other central neurodegenerations.

The review article by Stassart et al. provides an excellent summary of axon and glial cell biology and anatomy and how this interdependent relationship changes with disease (Stassart et al.). Similarly, axons and glial cells develop in concert, and undergo age-related changes that may predispose or even precipitate neurodegenerative diseases. The review by Salvadores et al. examines the impact of aging on the anatomy and function of axons (Salvadores et al.). Proper function of the axon depends on a well-order cytoskeleton. Cytoskeletal abnormalities are common to axon-centric diseases and toxic neuropathies, with specific chronic conditions such as tauopathies directly impacting axonal proteins. The review by Kneynsberg et al. examines the mechanisms by which tau, a microtubule associated protein, contributes to axon degeneration and disease (Kneynsberg et al.).

Another area of axonopathy research that has received a great deal of attention recently is chemotherapy-induced peripheral neuropathy (CIPN), a common side-effect of cancer treatment that decreases patient quality-of-life and is a major factor limiting drug dosage (Argyriou et al., 2014). This is a major concern clinically, but these drugs also provide tools for understanding axon degeneration and the normal cellular functions that are required to maintain healthy axons. The review by Fukuda et al. examines the cellular mechanisms underlying CIPN, which extend well beyond the impact of these drugs on the microtubule cytoskeleton (Fukuda et al.).

Axon degeneration is an active process, and this is most clearly demonstrated in Wallerian degeneration, which involves the fragmentation and disintegration of an axon distal to the site of an injury. Genetic studies have identified a spontaneous gain-of-function mouse mutation, Wallerian Degeneration Slowed (*WLD*^s^), as well as recessive loss-of-function mutations in *Sarm1* as mutations that protect against Wallerian degeneration (Mack et al., 2001; Osterloh et al., 2012). Both *WLD*^s^ and loss of *Sarm1* lead to increased NAD^+^ levels locally in the axon, which in turn protects axons from degeneration. The therapeutic potential of this pathway is demonstrated in the original research article by Williams et al. which shows that nicotinamide and *WLD*^s^ act in concert to prevent retinal ganglion cell axon loss in glaucoma (Williams et al.).

Like NAD^+^ levels, local ATP and Ca^++^ homeostasis in axons depends on healthy mitochondria, which traffic through, and are localized in axons. Mutations affecting mitochondrial fusion, fission, and metabolism lead to an assortment of axonal defects and are also important in regeneration. The original research article by Knowlton et al. describes the relationship between mitochondrial function and the capacity for axon regeneration in *C. elegans* (Knowlton et al.). The review by Inman et al. examines how metabolic vulnerability specifically in axons and myelinating glial cells can contribute to axon loss in glaucoma (Inman and Harun-Or-Rashid). In contrast to metabolic issues, the original research article by Rogers et al. suggests that impaired mitophagy in nerve terminals contributes to the die-back neuropathy of motor neurons seen in mouse models of ALS (Rogers et al.). The need to maintain healthy mitochondria far from the cell body is a recurring theme in many neurodegenerative diseases. This is one argument favoring local protein synthesis in axons, which remains a controversial topic in neurobiology. The state of this question is addressed in the review by Spaulding and Burgess, including recent *in vivo* profiling studies of ribosome-associated mRNAs isolated from axons of the adult mouse visual system that indicate robust levels of translation, particularly of mitochondrial components (Spaulding and Burgess).

Axonopathy is often considered in the context of peripheral motor and sensory neurons, given their length, the presence of diseases that specifically affect these systems, and their sensitivity to challenges such as chemotherapy drugs or metabolic disorders such as diabetes. However, these characteristics are not limited to the peripheral nervous system. Many of the papers in this research topic focus on glaucoma, a neuropathy affecting axons of the optic nerve, one of the few central nervous system components outside of the brain and spinal cord. Glaucoma shares commonalities with other central neurodegenerations such as Alzheimer's, Parkinson's, and Huntington's diseases, often exhibiting comorbidity with those conditions, as well as exhibiting similar mechanisms with these and other axonopathies (Conforti et al., 2007).

Stresses such as hypoxia and oxidative stress arising from vascular dysfunction contribute to the pathogenesis of glaucoma, as described in the original research article by Chidlow et al. As in the degenerating brain, neuroinflammation plays a sizable role in glaucomatous neurodegeneration. One of this topic's original research articles, the loss of the pleiotropic cytokine IL-6 is shown to protect axons in glaucoma (Echevarria et al.). The mechanism underlying this protection remains unclear, but changes in axonal transport appear to be separated from changes in axon integrity, possibly separating these features in this model. Another original research article shows that more conventional inflammatory pathways may also contribute to glaucoma (Lambert et al.). Treatment with the synthetic steroid HE3286 reduced axonopathy in a rodent microbead occlusion model of glaucoma, possibly through its proposed targets of MAPK/ERK/NFκb signaling. The recurring mechanisms of axonal transport and cytoskeletal abnormalities are also in play in the axonopathy of glaucoma, as shown in the original research articles by Breen et al. and Wilson et al. Finally, the differential sensitivity of neurons to degeneration is a common yet puzzling feature of a variety of diseases and neurotoxic conditions. The review by Vidal-Sanz et al. describes the differential responses of different retinal ganglion cell populations in animal models exhibiting either ocular hypertension or optic nerve injury (Vidal-Sanz et al.). Together, these papers highlight the parallels of glaucoma and other diseases in which axonopathy is a key pathophysiological sequela.

Although the mechanisms that lead to axon degeneration may be shared across a range of diseases, they encompass a wide range of biological processes including cytoskeleton, transport, metabolism, translation, and inflammation. Perhaps this reflects the number of things that normally have to go correctly to actually preserve an axon, which in turn, could explain why axonopathy is often the harbinger of degeneration. Defining these processes is a challenge and predicts that there will not be a single “magic bullet” to correct all axonopathies, but the rapidly increasing depth of our knowledge concerning the functions required to maintain an axon will ultimately help in understanding how to prevent degeneration or even to promote regeneration in these diseases in the future.

## Author contributions

All authors listed have made a substantial, direct and intellectual contribution to the work, and approved it for publication.

### Conflict of interest statement

The authors declare that the research was conducted in the absence of any commercial or financial relationships that could be construed as a potential conflict of interest.
